# Preliminary Ultrasonographic Optic Nerve Sheath Diameter Values in Clinically Healthy Adult Rahmani Sheep

**DOI:** 10.1155/vmi/1112219

**Published:** 2026-10-09

**Authors:** Alaa Alrajab, Mohamed Marzok, Adel Almubarak, Zakaria Al Mohamad, Mohamed El-Sherif

**Affiliations:** ^1^ Department of Clinical Studies College of Veterinary Medicine King Faisal University Al-Ahsa 31982 Saudi Arabia kfu.edu.sa; ^2^ Department of Surgery Faculty of Veterinary Medicine New Valley University Al-Kharga 27511 New Valley Egypt nv.aun.edu.eg

**Keywords:** intracranial pressure, neurological disease, optic nerve, small ruminant, transpalpebral, ultrasonography

## Abstract

**Background and Aims:**

Sheep reared in tropical and subtropical areas may develop neurological disorders associated with infectious, parasitic, metabolic, or nutritional diseases. Some of these disorders may be accompanied by increased intracranial pressure (ICP). Direct ICP measurement methods are invasive, and technically unavailable in clinical field settings. Ultrasonographic measurement of optic nerve sheath diameter (ONSD) has been reported as a noninvasive method of ICP indicator in humans and animal species. However, baseline ONSD values for clinically healthy sheep are not yet reported. Therefore, this pilot field study aimed to determine preliminary transpalpebral ultrasonographic ONSD values in clinically healthy adult Rahmani sheep.

**Methods:**

Sixty clinically healthy adult Rahmani sheep, including 30 males and 30 nonpregnant females, were examined under field conditions. All animals underwent physical, neurological, hydration, external ocular, and direct ophthalmoscopic examination before inclusion. Animals with systemic illness, dehydration, ocular disease, or any clinical sign suggestive of neurological dysfunction or raised ICP were excluded. Bilateral transpalpebral ultrasonography was performed in conscious standing sheep without sedation using a 10‐MHz linear probe. ONSD was measured at 3 mm posterior to the posterior retinal surface in the transverse plane. Because direct ICP measurement was not available and was not applicable under field conditions, ICP was not measured.

**Results:**

Transpalpebral ultrasonography was feasible in all examined sheep and was completed without sedation or adverse events. Mean ± SD ONSD was 4.65 ± 0.07 mm in the left eye and 4.70 ± 0.06 mm in the right eye, with no significant laterality difference (*p* = 0.34). No significant difference in animal‐level mean ONSD was detected between males and nonpregnant females (*p* = 0.23). The preliminary animal‐level ONSD interval was 4.55–4.77 mm. Inter‐ and intraobserver agreement was excellent (ICC = 0.93 and 0.94, respectively).

**Conclusions:**

Transpalpebral ultrasonography allowed feasible and repeatable measurement of ONSD in conscious, standing adult Rahmani sheep under field conditions. This pilot study provides preliminary baseline ONSD values in clinically healthy Rahmani sheep without detectable clinical evidence of disease.

**Limitations:**

Because ICP was not directly measured, these values should not be interpreted as diagnostic thresholds for intracranial hypertension. They may serve as foundational data for future studies comparing healthy sheep with sheep affected by neurological disease and for studies validating ONSD against direct ICP measurement when feasible.

## 1. Introduction

Elevated intracranial pressure (ICP) is an important pathophysiological condition that may occur secondary to infectious, parasitic, inflammatory, traumatic, metabolic, or space‐occupying intracranial disorders [1–4]. Sheep in tropical and subtropical regions are affected by various infectious diseases, including leptospirosis, listeriosis, and coenurosis, and noninfectious conditions such as magnesium and vitamin A or B deficiencies. These disorders are frequently associated with neurological dysfunction and depending on the underlying pathology may be associated with increased ICP [5–8]. Direct ICP measurement using ventricular catheterization, intraparenchymal probes, or epidural sensors remains the reference standard for ICP assessment. Nevertheless, these techniques are invasive, require specialized equipment and technical expertise, and are not suitable for routine use in conscious sheep under field conditions [9–11].

The optic nerve is surrounded by meninges, and the perioptic subarachnoid space communicates anatomically with the intracranial subarachnoid space. Therefore, increases in cerebrospinal fluid pressure may be transmitted to the retrobulbar optic nerve sheath, leading to sheath distension. This anatomical relationship provides the physiological basis for using optic nerve sheath diameter (ONSD) as a potential indirect marker of ICP changes [12–14].

On ultrasonographic assessment, the ONSD is measured 3 mm posterior to the globe. This standardized retrobulbar measurement site is adopted in human and veterinary studies [15–18].

In human medicine, ultrasonographic ONSD measurement has been widely investigated as a rapid, noninvasive method for detecting raised ICP [19, 20]. Experimental and clinical veterinary studies in species such as dogs, cats, pigs, and horses have also supported the feasibility of ONSD assessment. However, ONSD values and diagnostic thresholds are species‐specific and protocol‐dependent. Therefore, thresholds reported in humans or companion animals cannot be directly extrapolated to sheep [15, 16, 21–26].

Published data on ultrasonographic ONSD in sheep remain limited. Baseline values have not been reported in healthy adult Rahmani sheep, limiting their use in studies of neurological disease and ICP‐related changes [11].

We hypothesized that transpalpebral ultrasonography could provide feasible and repeatable ONSD measurements in conscious, standing adult Rahmani sheep under field conditions, and that clinically healthy sheep without signs suggestive of raised ICP would show a narrow preliminary range of ONSD values. The current pilot study aimed to determine preliminary baseline transpalpebral ultrasonographic ONSD values in clinically healthy adult Rahmani sheep. These data are intended to support future studies comparing healthy sheep with sheep showing neurological signs and, when feasible, validating ONSD against direct ICP measurement.

## 2. Materials and Methods

This was a prospective, cross‐sectional observational pilot field study designed to determine preliminary transpalpebral ultrasonographic ONSD values in clinically healthy adult Rahmani sheep. The study was not designed to validate ONSD against ICP because direct ICP measurement was not available and was not applicable under the field conditions of this work. Therefore, animals were selected based on field applicable clinical criteria indicating apparent health and absence of signs suggestive of raised ICP.

### 2.1. Animals

The animals were recruited from a single privately owned Rahmani sheep farm. Sixty clinically healthy adult Rahmani sheep were enrolled, including 30 males and 30 nonpregnant females. A total of 120 eyes were examined. Mean age was 27.3 months, with a range of 19–36 months, and mean body weight was 59.6 kg, with a range of 51–68 kg. Nonpregnancy in female sheep was confirmed by transabdominal ultrasonography on the day of examination before inclusion.

Inclusion required apparent clinical health based on field‐applicable physical examination, hydration assessment, external ocular examination, bilateral direct ophthalmoscopy, and neurological observation. Sheep were excluded if they showed fever, depression, abnormal appetite, dehydration, abnormal mucous membranes, respiratory distress, diarrhea, systemic illness, ocular disease, orbital or periorbital abnormalities, corneal opacity, uveitis, glaucoma, optic disc abnormality, or any clinical sign suggestive of neurological dysfunction. Neurological exclusion signs included abnormal mentation, circling, head pressing, ataxia, recumbency, seizures, opisthotonus, abnormal menace response, abnormal pupillary light reflex, nystagmus, blindness, or cranial nerve deficits.

Systemic blood pressure was not measured; therefore, occult blood pressure–related effects on ONSD could not be excluded and were considered a study limitation.

### 2.2. Standardization of Examination Conditions

To minimize physiological and procedural variation, all ultrasonographic examinations were conducted between 6 and 9 a.m. before the first meal of the day. All sheep were examined in a standing position under gentle manual restraint by their shepherd. Sedation was not used. This approach was selected to preserve normal field conditions and to avoid potential sedative‐related effects on ICP, venous pressure, ocular parameters, or optic nerve sheath dynamics. Animals showing agitation or excessive movement were allowed to rest for five minutes before re‐examination.

### 2.3. Transpalpebral Ultrasonography

The transpalpebral approach was selected because it allows imaging through the closed eyelid, avoids direct corneal contact, requires only acoustic gel, and can be performed in conscious animals with minimal restraint. These characteristics make the technique suitable for field use in sheep, where hospital‐based imaging, anesthesia, and invasive monitoring are often unavailable.

### 2.4. Ultrasound and Transducer Settings

Ocular ultrasonography was performed using a wireless linear transducer operating at 10 MHz (C10RL, KontedMed, China). Imaging depth was set to 60 mm. Acoustic gel was applied to the closed upper eyelid. Probe pressure was minimized throughout the examination to avoid globe deformation and reduce the possibility of pressure‐related measurement artifacts. Images showing globe deformation, poor optic nerve visualization, or unclear sheath margins were excluded from measurement.

### 2.5. ONSD Measurement

Optic nerve was identified as a hypoechoic structure surrounded by the more echogenic optic nerve sheath complex. At 3 mm posterior to the retinal surface, calipers were placed on the outer contours of the optic nerve sheath, and ONSD was defined as the outer‐to‐outer transverse distance between these boundaries, measured perpendicular to the long axis of the optic nerve [15, 21, 27, 28]. For each eye, two qualifying images were measured, and the mean value was used for statistical analysis.

To reduce selection bias, the first two qualifying images for each eye were used rather than selecting subjectively optimal images. Images qualified for measurement when the optic nerve was visible for at least 3 mm posterior to the retinal surface, both optic nerve sheath margins were clearly delineated, and no globe deformation or acoustic shadowing artifact was present.

### 2.6. Reliability Assessment

For reliability assessment, 60 eyes were randomly selected. Two observers independently measured the same anonymized images while blinded to animal sex and to each other’s measurements. For intraobserver repeatability, one observer repeated the measurements after an interval without access to the first measurements.

### 2.7. Statistical Analysis

Continuous variables were assessed for distributional characteristics, including graphical evaluation and the Shapiro–Wilk test. Approximately normally distributed continuous data are presented as mean ± standard deviation (SD) with 95% confidence intervals (CIs) where appropriate. Nonnormally distributed continuous data are presented as median and interquartile range (IQR) or range, as appropriate.

Left‐ and right‐eye ONSD measurements were compared within animals using paired *t*‐test, with the paired difference and its 95% CI reported as an estimate of the magnitude and precision of the laterality effect. Sex‐related differences in animal‐level mean ONSD were assessed using an independent‐sample *t*‐test, with the between‐group mean difference and 95% CI reported. Associations between animal‐level mean ONSD and age and body weight were assessed using Pearson’s correlation coefficient when the corresponding assumptions were met, correlation coefficients are reported with 95% CIs.

Because measurements from both eyes of the same sheep are not statistically independent, preliminary ONSD intervals were calculated from the animal‐level mean ONSD. The 2.5th and 97.5th percentiles were used to estimate the preliminary interval, with bootstrap CIs calculated for the interval limits using 1000 resamples. These intervals were considered preliminary rather than definitive clinical reference intervals because of the limited sample size and single‐farm population.

Inter‐ and intraobserver agreement were assessed using two‐way mixed‐effects intraclass correlation coefficients for absolute agreement, with 95% CIs. Bland–Altman analysis was used to quantify mean measurement bias and 95% limits of agreement.

All hypothesis tests were two‐sided, and the a priori level of statistical significance was set at *α* = 0.05. Statistical analyses were performed using SPSS V. 26.0, IBM, USA.

## 3. Results

### 3.1. Study Population

All 60 Rahmani sheep completed the ultrasonographic examination successfully. A total of 120 eyes were available for analysis. No animal was excluded after enrollment because of inadequate image quality or procedural intolerance. No adverse events were observed during or after examinations. The study population included 30 males and 30 nonpregnant females. Demographic characteristics are presented in Table 1.

**TABLE 1:** Demographic characteristics of the study cohort (*n = 60*).

Variable	Males *(n = 30)*	Nonpregnant females *(n = 30)*
Age (months)	26.8 ± 4.2	27.1 ± 4.0
Body weight (kg)	61.2 ± 4.1	58.0 ± 3.9
Eyes examined (*n*)	60	60

*Note:* Values are mean ± SD.

### 3.2. Technique Feasibility

Transpalpebral ultrasonography was feasible in all examined animals. The procedure was completed in conscious standing sheep using gentle manual restraint and without sedation. Representative images consistently demonstrated the posterior globe, the hyperechoic retinal surface, and the echogenic optic nerve sheath, supporting the feasibility of the technique under field conditions (Figure 1).

**FIGURE 1:**
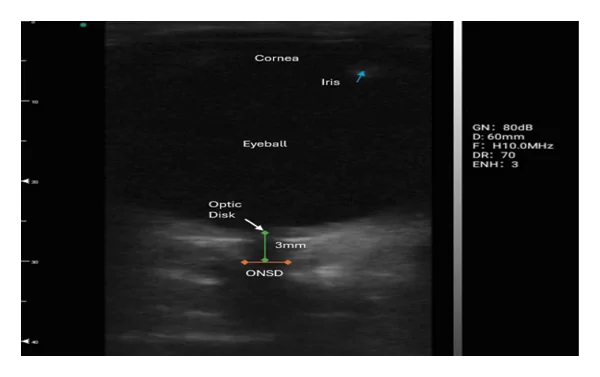
Transpalpebral B‐mode ultrasonographic measurement of optic nerve sheath diameter (ONSD) in a clinically healthy adult Rahmani sheep. ONSD was measured in the transverse plane at 3 mm posterior to the retinal surface, perpendicular to the long axis of the optic nerve, using outer‐to‐outer caliper placement at the visible optic nerve sheath boundaries. The optic disc, optic nerve, optic nerve sheath, and 3‐mm measurement position are indicated on the image.

### 3.3. Continuous Covariates

In the combined cohort, ONSD showed no meaningful correlation with age or body weight at either eye level or animal level. At the animal level, mean ONSD was not significantly correlated with age (Pearson’s *r* = 0.08, *p* = 0.54) or body weight (*r* = 0.11, *p* = 0.41). Similar results were obtained for the left eye (age: *r* = 0.07, *p* = 0.59; body weight: *r* = 0.10, *p* = 0.45) and right eye (age: *r* = 0.09, *p* = 0.49; body weight: *r* = 0.12, *p* = 0.36). Given the relatively narrow ranges of age and body weight in the study population, these findings indicate no detectable associations within the present cohort rather than evidence that age or body size does not influence ONSD in the broader sheep population.

### 3.4. ONSD Values and Reference Intervals

In the full cohort, mean ONSD was 4.65 ± 0.07 mm in the left eye and 4.70 ± 0.06 mm in the right eye, with no significant laterality difference. At the animal level, the preliminary reference interval for Rahmani sheep was 4.55–4.77 mm, with bootstrap confidence limits reflecting the limited sample size. Group‐specific animal‐level reference intervals were 4.55–4.76 mm for males and 4.58–4.81 mm for nonpregnant females (Table 2) (Figure 2).

**TABLE 2:** ONSD measurements by group in adult Rahmani sheep.

Measure	Value	
Left eye ONSD	4.65 ± 0.07 mm	No significant laterality difference versus right eye; paired test *p* = 0.34.
Right eye ONSD	4.70 ± 0.06 mm	No significant laterality difference versus left eye; paired test *p* = 0.34.
Males	Animal‐level preliminary interval: 4.55–4.76 mm	30 males; 90% bootstrap CI for interval limits: lower 4.55–4.61 mm, upper 4.74–4.77 mm.
Nonpregnant females	Animal‐level preliminary interval: 4.58–4.81 mm	30 nonpregnant females; nonpregnancy was confirmed by transabdominal ultrasonography on the day of examination.
Combined groups	Animal‐level preliminary interval: 4.55–4.77 mm	60 sheep; 90% bootstrap CI for interval limits: lower 4.55–4.60 mm, upper 4.75–4.88 mm.

*Note:* Values are presented as mean ± SD unless otherwise stated. The animal‐level preliminary interval was calculated from the per‐animal mean ONSD to avoid pseudo‐replication from using both eyes of the same animal. Bootstrap CIs are 90% for the lower and upper interval limits and were based on 1000 resamples. Because the sample size was limited and animals were recruited from a single farm, these intervals should be interpreted as preliminary baseline values rather than definitive clinical reference intervals. No post hoc or Tukey adjustment was applicable because the study included only two sex groups.

Abbreviations: CI, confidence interval; ONSD, optic nerve sheath diameter.

**FIGURE 2:**
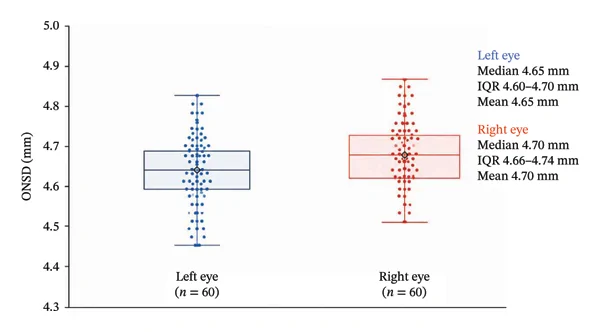
Distribution of optic nerve sheath diameter (ONSD) measurements in adult Rahmani sheep. Boxplots illustrating the distribution of ONSD measurements obtained from the left and right eyes of clinically healthy adult Rahmani sheep. The horizontal line within each box represents the median, the box indicates the interquartile range (IQR), and the whiskers extend to 1.5 × IQR. Individual points represent ONSD measurements from each eye, and diamonds indicate the mean value. The distributions demonstrate the narrow variability of ONSD measurements and the absence of marked outliers in this cohort.

### 3.5. Sex‐Related Comparison

No significant difference in animal‐level mean ONSD was detected. Animal‐level mean ONSD was 4.662 ± 0.054 mm (95% CI, 4.642 to 4.683 mm) in males and 4.659 ± 0.052 mm (95% CI, 4.639 to 4.678 mm) in nonpregnant females. The mean between‐group difference was 0.004 mm (95% CI, −0.024 to 0.031 mm, independent‐sample *t*‐test: *t* (58) = 0.26, *p* = 0.79). Group‐specific preliminary intervals are presented descriptively. Because the study included only two sex groups, this comparison represents a prespecified two‐group contrast rather than a post hoc multiple‐group comparison.

### 3.6. Reliability

Measurement agreement was excellent. Interobserver ICC was 0.93 (95% CI: 0.89–0.96), and intraobserver ICC was 0.94 (95% CI: 0.91–0.97), indicating high repeatability of the measurement protocol. The Bland–Altman analysis demonstrated a mean interobserver difference of 0.02 mm (95% limits of agreement: −0.09 to 0.13 mm) and a mean intraobserver difference of 0.01 mm (95% limits of agreement: −0.08 to 0.10 mm).

## 4. Discussion

This pilot field study provides preliminary transpalpebral ultrasonographic ONSD values in clinically healthy adult Rahmani sheep examined under field conditions. ONSD measurement was feasible in conscious, standing sheep without sedation, image acquisition was achieved using minimal restraint, and measurement repeatability was high. These findings support the practical feasibility of the transpalpebral approach for ONSD assessment in sheep under field conditions.

Importantly, ICP was not directly measured in this study. Accordingly, the reported ONSD values represent preliminary baseline measurements in sheep without detectable neurological, systemic, ocular, or hydration abnormalities and should not be interpreted as validated reference values for normal ICP or as diagnostic thresholds for intracranial hypertension. Establishing such diagnostic applications will require studies comparing ONSD with direct ICP measurements and/or ONSD measurements in sheep with intracranial disease.

The clinical relevance of this work lies in its potential field application. Sheep raised under tropical and subtropical conditions may develop neurological signs associated with infectious, parasitic, metabolic, nutritional, or inflammatory disorders. In many field situations, advanced imaging and invasive ICP monitoring are not available. Therefore, a portable, rapid, noninvasive method applicable to conscious, standing sheep may have value in future field‐based neurological assessment protocols.

The transpalpebral approach was practical in the present study because it allowed imaging through the closed eyelid, avoided direct corneal contact, and did not require sedation. Avoiding sedation is an important advantage in field studies because sedative and anesthetic drugs may influence cardiovascular, respiratory, venous, ocular, and possibly intracranial physiological variables. The ability to perform the examination in standing sheep with minimal restraint supports the suitability of this technique for field‐based veterinary work.

The 3‐mm posterior measurement site was selected because it is widely used in human and veterinary ONSD protocols. This site is considered sensitive to ICP‐related sheath distension because the anterior retrobulbar optic nerve sheath is more distensible than more posterior segments. Therefore, adopting this measurement site improves methodological comparability with previous ONSD studies in humans and animals [15–18]. Accordingly, in the present study, the 3‐mm measurement point was adopted as a standardized methodological reference for ONSD assessment rather than as a sheep‐specific diagnostic standard.

The ONSD values observed in Rahmani sheep differ from values reported in other species. Such differences are expected because ONSD is influenced by species‐specific anatomy, body size, imaging protocol, measurement site, and possibly skull and orbital conformation. Therefore, ONSD values and diagnostic thresholds developed in humans, dogs, cats, pigs, or horses cannot be directly transferred to sheep. This supports the need for ovine‐specific baseline data before clinical interpretation can be attempted [15, 23–26, 29].

In humans, normative ONSD values and proposed thresholds for raised ICP vary across studies and imaging protocols. A recent scoping review indicated that ONSD generally correlates positively with invasively measured ICP in traumatic brain injury patients, with values above approximately 5.0 mm often associated with ICP ≥ 20 mmHg [17]. However, these human thresholds should not be applied to sheep. The present sheep values fall within a numerical range that overlaps partly with human values, but this overlap should be considered coincidental and anatomically species‐dependent rather than evidence of similar ICP physiology.

Measurement reliability was high, with an interobserver ICC of 0.93 and an intraobserver ICC of 0.94. The small inter‐ and intraobserver mean differences and narrow limits of agreement further support the repeatability of the measurement protocol under the conditions of this study. Similar reproducibility has been reported in transpalpebral ONSD studies in dogs and cats [15, 23]. The absence of clinically relevant systematic bias between observers further supports the consistency of the protocol.

The narrow between‐animal variability observed in the present study may reflect the biological homogeneity of the examined population. All animals belonged to a single breed, were recruited from one farm, and represented a relatively narrow age and body‐weight range. Although the ultrasound system had finite axial resolution, measurement of clearly defined hyperechoic sheath margins may still allow consistent caliper placement when image quality is adequate. Nevertheless, the narrow SDs should be interpreted cautiously because the current population may not represent the broader Rahmani sheep population or other ovine breeds [23, 26].

No meaningful association was detected between ONSD and age or body weight in this cohort. However, the animals represented a relatively narrow age and body‐weight range and belonged to a single breed. Therefore, the absence of a detectable association should not be interpreted as proof that age, body size, breed, or skull conformation have no effect on ONSD in sheep. Larger multibreed studies covering wider physiological ranges are required to evaluate these possible effects. Future studies may also consider body weight‐ or body surface area‐normalized ONSD values, especially when comparing breeds or animals with wide body‐size variation.

No significant difference in ONSD was detected between males and nonpregnant females in the present study. Nevertheless, reproductive cycle stage and reproductive hormone concentrations were not recorded in the female sheep. Therefore, possible effects of estrous cycle stage or hormonal status on ONSD cannot be excluded. Previous companion‐animal studies suggest that reproductive or hormonal status may influence ocular or periocular physiological variables, including intraocular pressure and ONSD‐related parameters [30–33]. Future studies in sheep should document pregnancy status, estrous cycle stage, and, when possible, reproductive hormone profiles.

### 4.1. Limitations and Future Work

First, ICP was not directly measured; therefore, the reported ONSD values cannot be interpreted as validated indicators of normal ICP or as diagnostic thresholds for intracranial hypertension. Classification of the enrolled sheep as clinically healthy was based on the absence of detectable neurological, systemic, ocular, and hydration abnormalities under field conditions rather than direct confirmation of normal ICP. Second, animals were recruited from a single farm and represented one breed with a relatively narrow age and body‐weight range. Third, systemic blood pressure, intraocular pressure, laboratory screening, cerebrospinal fluid analysis, and advanced neuroimaging were not performed. Fourth, reproductive cycle stage in nonpregnant females was not recorded. Fifth, no sheep with neurological disease were included. Consequently, the findings should be considered preliminary baseline data requiring confirmation in larger, multicenter, multibreed studies and validation in sheep with suspected or confirmed intracranial disease.

Future research should proceed in two main directions. First, larger studies should confirm baseline ONSD values in different ovine breeds, age groups, body sizes, reproductive stages, and management systems. Second, clinical and experimental studies should compare ONSD values between clinically healthy sheep and sheep with neurological disease, ideally including concurrent direct ICP measurement when ethically and technically feasible. Such studies are necessary before transpalpebral ONSD ultrasonography can be used as a diagnostic or monitoring tool for raised ICP in sheep.

## 5. Conclusion

Transpalpebral ultrasonography allowed feasible and repeatable measurement of ONSD in conscious, standing adult Rahmani sheep under field conditions. This pilot study provides preliminary baseline ONSD values in clinically healthy Rahmani sheep with no detectable signs of neurological, systemic, ocular, or hydration abnormalities. Because ICP was not directly measured, these values should not be interpreted as diagnostic thresholds for intracranial hypertension. They provide a foundation for future studies comparing healthy sheep with sheep affected by neurological disease and for validation against direct ICP measurement when technically feasible.

## Author Contributions

Alaa Alrajab contributed to data curation and analysis and the provision of research facilities. Mohamed Marzok contributed to the conception of the study, study design, supervision, data interpretation, and manuscript drafting and revision. Adel Almubarak contributed to study design, field coordination, and critical revision of the manuscript. Zakaria Al Mohamad contributed to animal examination, data collection, and manuscript revision. Mohamed El‐Sherif contributed to conceptualization, study design, ultrasonographic examination, data acquisition, image handling, manuscript drafting, and revision. Alaa Alrajab and Mohamed El‐Sherif share first authorship.

## Funding

This work was supported through the Annual Funding track by the Deanship of Scientific Research, Vice Presidency for Graduate Studies and Scientific Research, King Faisal University, Saudi Arabia, Protocol Number (KFU265664).

## Disclosure

All authors have read and approved the final version of the manuscript. The funding source had no role in the study design, collection, analysis, or interpretation of the data preparation or the decision to submit the manuscript for publication.

The manuscript guarantor affirms that this manuscript is an honest, accurate, and transparent account of the study being reported; that no important aspects of the study have been omitted; and that any discrepancies from the study as planned have been explained.

## Ethics Statement

The current study was reviewed, permitted, and approved by the Research Ethics Committee (REC) of King Faisal University, KSA. All methods were carried out in accordance with relevant guidelines and regulations. Written consents were obtained from farm owners before initiation of the experiment. All methods are reported in accordance with the ARRIVE guidelines [34].

## Conflicts of Interest

The authors declare no conflicts of interest.

## Data Availability

The data that support the findings of this study are available from the corresponding authors upon reasonable request.
